# Biosyntheses characterization of alkaloids and flavonoids in *Sophora flavescens* by combining metabolome and transcriptome

**DOI:** 10.1038/s41598-021-86970-0

**Published:** 2021-04-01

**Authors:** Guangfei Wei, Yongzhong Chen, Xiaotong Guo, Jianhe Wei, Linlin Dong, Shilin Chen

**Affiliations:** 1 Key Laboratory of Beijing for Identification and Safety Evaluation of Chinese Medicine, Institute of Chinese Materia Medica, China Academy of Chinese Medical Sciences, Beijing, 100700 China; 2 College of Agriculture, Ludong University, Yantai, 264025 China; 3 Hainan Provincial Key Laboratory of Resources Conservation and Development of Southern Medicine, Hainan Branch of the Institute of Medicinal Plant Development, Chinese Academy of Medical Sciences and Peking Union Medical College, Haikou, 570311 China; 4 Present Address: No.16 Nanxiaojie, Dongzhimennei Ave., Beijing, 100700 China

**Keywords:** Molecular biology, Chemistry, Biosynthesis, Metabolic pathways, Metabolomics

## Abstract

*Sophora flavescens* are widely used for their pharmacological effects. As its main pharmacological components, alkaloids and flavonoids are distributed in the root tissues wherein molecular mechanisms remain elusive. In this study, metabolite profiles are analyzed using metabolomes to obtain biomarkers detected in different root tissues. These biomarkers include alkaloids, phenylpropanoids, and flavonoids. The high-performance liquid chromatography analysis results indicate the differences in principal component contents. Oxymatrine, sophoridine, and matrine contents are the highest in the phloem, whereas trifolirhizin, maackiain, and kushenol I contents are the highest in the xylem. The transcript expression profiles also show tissue specificity in the roots. A total of 52 and 39 transcripts involved in alkaloid and flavonoid syntheses are found, respectively. Among them, the expression levels of LYSA1, LYSA2, AO2, AO6, PMT1, PMT17, PMT34, and PMT35 transcripts are highly and positively correlated with alkaloids contents. The expression levels of 4CL1, 4CL3, 4CL12, CHI5, CHI7, and CHI9 transcripts are markedly and positively correlated with flavonoids contents. Moreover, the quantitative profiles of alkaloids and flavonoids are provided, and the pivotal genes regulating their distribution in *S. flavescens* are determined*.* These results contribute to the existing data for the genetic improvement and target breeding of *S. flavescens*.

## Introduction

*Sophora flavescens*, which belong to Leguminous family, present remarkable antiviral effects and are commonly used in Traditional Chinese Medicine^1^. The output value of Chinese medicine preparation contaning *S. flavescens* has exceeded 500 million RMB (Renminbi), such as compound Radix Sophora Flavescentis Injection, Matrine Injection, Fuyankang Tablets, and Zhidai Tablets^2^. A recent study showed that Matrine Sodium Chloride Injection had an evident therapeutic effect, and the inhibition rate of lung index in the model group was as high as 86.86%^3^. Another study showed that Matrine and Sodium Chloride Injection effectivity rate of coronavirus disease 2019 (COVID-19) as a clinical drug was 100% among 40 patients^4^.

The main active components of *S. flavescens* are alkaloids and flavonoids. The alkaloids mainly include matrine, oxymatrine, sophorine, and oxysophoridine. Flavonoids mainly include trifolirhizin, maackiain, kushenol I, kurarinone, and sophoraflavanone G^5,6^. The contents of matrine and oxymatrine have similar distribution rules in the root of *S. flavescens* as follows: lower lateral root > upper lateral root > main root > underground stem > stem bud^7^. The alkaloids contents in the root tissues is as follows: phloem > xylem > pith > cork layer^8^. The above studies preliminarily explained the differences of principal component contents, which provided a basis for the rational cultivation of *S. flavescens*. However, the research on the whole metabolic spectrum in the root tissues of *S. flavescens* is not systematic, and the complex biosynthesis mechanism of active components is weak.

Metabolomics have been widely used to study the distribution differences of active ingredients in medicinal plants^9,10^. In an early metabolomics study, 24 and 88 potential biomarkers (importance in projection [VIP] > 1) were found in the root tissues of *Panax notoginseng* and *Panax quinquefolius*, respectively^11^. More than 200 compounds have been isolated and identified from *Sophorae* Radix, including alkaloids, flavonoids, terpenoids, and other compounds^12^. Their composition and content vary between the organs of *Sophorae* Radix, such as the roots, stems, leaves, flowers, and seeds^13^. However, the reports on the distribution of alkaloids and flavonoids components in the root tissues of *S. flavescens* are rare. Therefore, the difference in the profiles of alkaloids and flavonoids components in the root tissues of *S. flavescens* should be established for the targeted breeding of this species.

The biosynthesis of active ingredients in medicinal plants is often related to the synergistic expression and regulation of key enzyme genes. Transcriptomes have been used to study the transcription and expression levels of genes in medicinal plants^14^. For example, a total of 749 ginsenoside biosynthetic enzyme genes, together with 12 good pleiotropic drug resistance genes related to ginsenoside transport, were identified from the adventitious roots of *Panax ginseng*^15^. In *Salvia miltiorrhiza*, 6358 genes, 70 transcription factors, and eight cytochromes P450 exhibited differential expressions^16^. In *Ginkgo biloba*, 66 unigenes responsible for terpenoid backbone biosynthesis were found. Approximately 12 up-regulated unigenes were involved in the biosynthesis of ginkgolide and bilobalide^17^. However, gene discovery and candidate genes involved in alkaloids and flavonoids in *S. flavescens* are still limited. In the absence of genome-wide studies on *S. flavescens*, transcriptional expression profiling can be used to rapidly identify gene expression, which is suitable for establishing the synergistic expression differences of the root tissues in this species*.*

In the current study, some genes participating in the alkaloid and flavonoid syntheses are hypothesized to regulate the distribution of alkaloids and flavonoids in the root tissues of *S. flavescens*. Firstly, metabolomics are used to establish the metabolic spectrum for revealing the distribution of alkaloids and flavonoids. Transcriptomes are also used to determine gene expression profiles for identifying expressed genes related to the alkaloid and flavonoid syntheses. This study willl analyze the biological mechanisms of the alkaloid and flavonoid syntheses in *S. flavescens*, and provide a basis for the genetic improvement and target breeding of this species.

## Results

### Metabolomic profiles in the root tissues of *S. flavescens*

**Figure 1:**
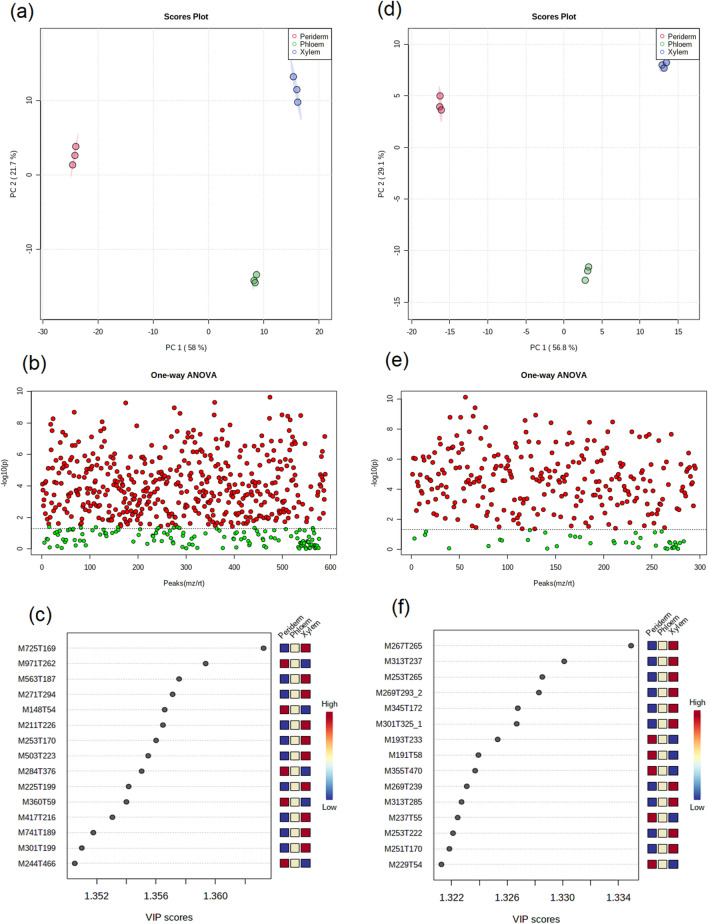
Metabolomic analysis of the components in the root tissues of *S. flavescens*. (**a**) PCA score plots of positive ion mode. (**b**) One-way ANOVA of the positive ion mode. (**c**) Variable importance in the projection of positive ion mode. (**d**) PCA score plots of negative ion mode. (**e**) One-way ANOVA of negative ion mode. (**f**) Variable importance in the projection of negative ion mode.

The chemical components in three root tissues of *S. flavescens* were determined using the ultra-high-performance liquid chromatography-mass spectrometry (UPLC-MS) analysis. In positive ion mode, 13,184 components were detected, and 589 components were identified (Supplemental file 2: Dataset S1). The principal component analysis (PCA) results revealed a clear separation between the three root tissues of *S. flavescens* in positive ion mode (Fig. 1a). A total of 387 potential biomarkers were detected in positive ion mode through one-way analysis of variance (ANOVA; false discovery rate [FDR] ≤ 0.05; Fig. 1b and Supplemental file 2: Dataset S1). M725T169 (3,3′,4′-Trihydroxyflavone-3-O-[a-l-rhamnopyranosyl-(1-&gt;2)[a-l-rhamnopyranosyl-(1-&gt;6)]-b-d-glucopyranoside]), M563T187 (Chrysin 7-[rhamnosyl-(1-&gt;4)-glucoside]), M271T294 (Genistein), M211T226 ((.+ /-.)7-epi-Jasmonic acid), M253T170 (Ser-Phe), M503T223 (6″-O-Malonyldaidzin), M225T199 (Methyl jasmonate), M417T216 (Daidzin), M741T189 (Kaempferol-3-O-robinoside-7-O-rhamnoside), and M301T199 (Chrysoeriol) were the most abundantly present in the xylem tissue (VIP > 1; Fig. 1c and Supplemental file 2: Dataset S1).

In negative ion mode, 11,101 components were detected, among which 297 components were identified (Supplemental file 2: Dataset S2). The PCA results also revealed a clear separation between the three root tissues of *S. flavescens* (Fig. 1d). A total of 257 potential biomarkers were detected in negative ion mode through ANOVA analysis (FDR ≤ 0.05; Fig. 1e and Supplemental file 2: Dataset S2). M267T265 (Formononetin), M313T237 (Velutin), M253T265 (Daidzein), M269T2932 (Apigenin), M345T172 (Propylthiouracil N-.beta.-D-glucuronide), M301T3251 (Moracin M), M269T239 (Aloe-emodin), M313T285 (Velutin), and M251T170 (Ser-Phe) were the most abundantly present in the xylem tissue (VIP > 1; Fig. 1f and Supplemental file 2: Dataset S2). These data showed that metabolites existed differences in the root tissues of *S. flavescens*.

### Distribution of alkaloids and flavonoids in the root tissues of *S. flavescens*

**Figure 2:**
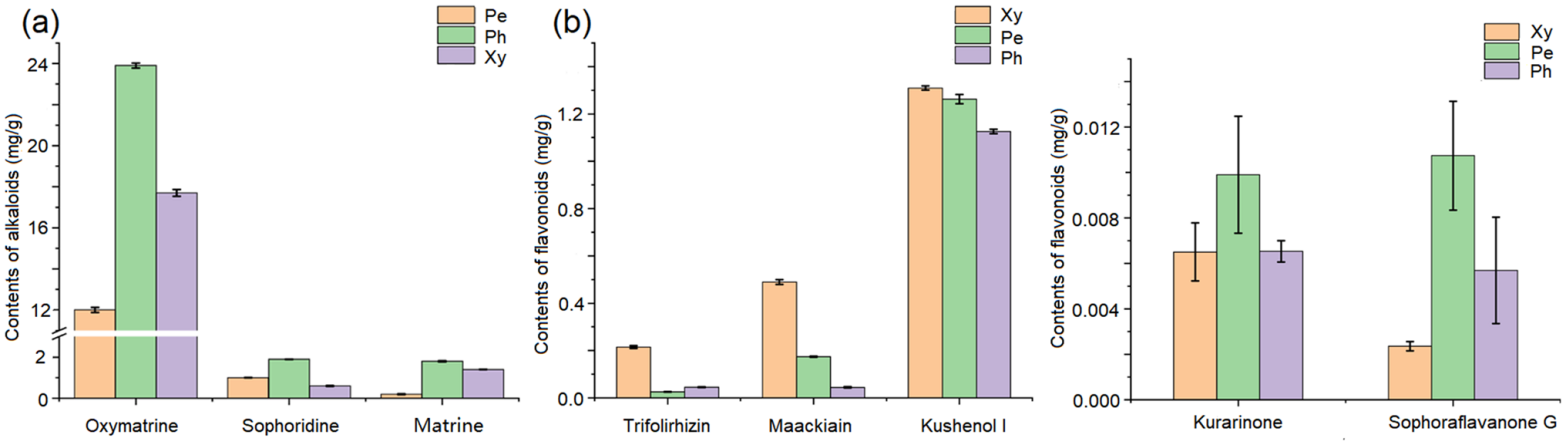
Contents of alkaloids and flavonoids in the root tissues of *S. flavescens*. (**a**) Contents of three alkaloids. (**b**) Contents of five flavonoids. Pe, Ph, and Xy represent the periderm, phloem, and xylem, respectively.

The contents of the three alkaloids (oxymatrine, sophoridine, and matrine) and five flavonoids (trifolirhizin, maackiain, kushenol I, kurarinone, and sophoraflavanone G) were detected through HPLC (Fig. 2). The contents of the three alkaloids were the highest in the phloem (23.93, 1.88, and 1.83 mg/g, respectively), followed by the xylem (17.73, 0.56, and 1.40 mg/g, respectively) and periderm (11.97, 1.04, and 0.21, respectively; Fig. 2a). The contents of trifolirhizin, maackiain, and kushenol I were the highest in the xylem (0.21, 0.49, and 1.31 mg/g, respectively). Finally, kurarinone and sophoraflavanone G contents were the highest in the periderm (0.01 and 0.01 mg/g, respectively; Fig. 2b). These results showed that alkaloids and flavonoids existed distribution differences in the root tissues of *S. flavescens*.

### Transcriptome profiles in the root tissues of *S. flavescens*

Illumina Hiseq paired-end sequencing technology was used to analyze the transcriptome in the root tissues of *S. flavescens* (Table 1). The average raw reads was 7.61 G, and the average clean reads was 6.81 G after filtered by fqtrim software. The percentages of Q20, Q30, and GC were 98.35%, 94.89%, and 45.83%, respectively. All 296,523,264 high-quality 150 bp clean reads were used for de novo assembly, and in total 35,012 contigs > 500 bp in length were obtained (Table 2). Then, a total of 58,327 genes were assembled, with an N50 contig size of 1,237. Annotation was also performed on the basis of the sequence similarity searches with a cutoff E-value of 10^−5^ against public databases, including the GO, Kyoto Encyclopedia of Genes and Genomes (KEGG), Pfam, SwissProt, eggNOG, and Nr databases, to investigate the function of assembled unigenes (Table 3). A total of 24,549 (66.63%), 19,056 (51.72%), 21,697 (58.89%), 20,069 (54.47%), 27,094 (73.54%), and 27,647 (75.04%) unigenes had significant matches with the GO, KEGG, Pfam, SwissProt, eggNOG, and NR databases, respectively.

**Table 1:** Statistics of transcriptome data for *S. flavescens.*

Samples	Raw reads	Raw bases	Clean reads	Clean bases	Q20%	Q30%	GC%
Periderm 1	56,056,078	8.41G	54,669,246	7.55G	98.69	95.55	45.28
Periderm 2	51,184,822	7.68G	49,657,472	6.82G	98.17	94.45	47.27
Periderm 3	45,266,188	6.79G	43,970,534	6.07G	98.37	95.16	46.50
Phloem 1	49,555,614	7.43G	48,015,280	6.60G	98.23	94.65	46.44
Phloem 2	63,603,050	9.54G	61,977,910	8.54G	98.31	94.78	45.33
Phloem 3	50,063,620	7.51G	48,836,916	6.73G	98.37	94.93	45.47
Xylem 1	45,639,898	6.85G	44,549,806	6.14G	98.35	94.89	45.67
Xylem 2	47,552,830	7.13G	46,387,724	6.39G	98.30	94.75	45.15
Xylem 3	47,837,988	7.18G	46,720,006	6.44G	98.34	94.87	45.34
Average	50,751,121	7.61G	49,420,544	6.81G	98.35	94.89	45.83

**Table 2:** Summary of the transcriptome data and the assembly results of *Sophora flavescens.*

Item	No. of sequences
High-quality reads	296,523,264
No. of contig > 500 bp	35,012
Total unigenes	58,327
N50 contig size (bp)	1,237
Total assemble bases (bp)	50,280,338

**Table 3:** The annotation of all unigenes in *Sophora flavescens.*

Total	Nr	Nt	Swiss-Prot	KEGG	COG	GO	Overall
84,408	68,720	49,929	52,118	52,176	28,431	42,535	69,951
100%	81.88%	61.29%	63.65%	63.54%	36.02%	52.80%	83.10%

### Gene expression profiles in the root tissues of *S. flavescens*

**Figure 3:**
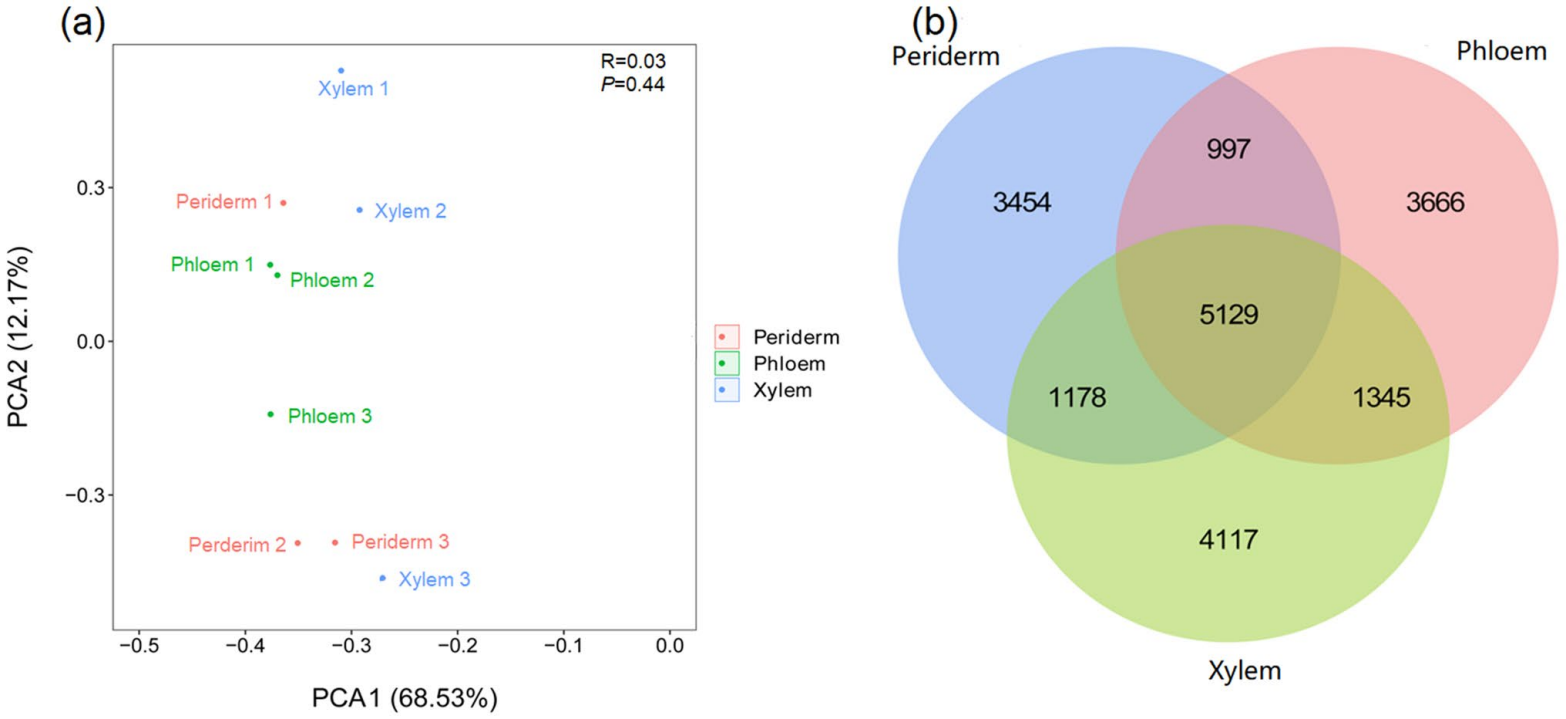
(**a**) Principal component analysis (PCA) and (**b**) Venn profiles in the roots of *S. flavescens*.

The PCA and Venn profiles were carried out to investigate the transcription distinction among the main root tissues in *S. flavescens* on the basis of the Fragments Per Kilobase of exon per Million fragments (FPKM) value (Fig. 3). The PCA results showed that the three root tissues existed slightly difference (Fig. 3a). The Venn results showed that 5129 unigenes were shared and expressed among the three tissues. A total of 3454, 3666, and 4117 unigenes were explicitly defined in the periderm, phloem, and xylem, respectively (Fig. 3b). With the comparison between the periderm and the phloem and xylem, 82 (Up: Down, 63: 19) and 654 (Up: Down, 454: 200) DEGs were found, respectively (Supplemental file 1: Figure S2). With the comparison between the phloem and the xylem, 186 (Up: Down, 45: 141) DEGs were found. These results showed that the unigenes in the root tissues of *S. flavescens* had different expression levels.

### Co-expression analysis of the transcripts and active components in *S. flavescens*

**Figure 4:**
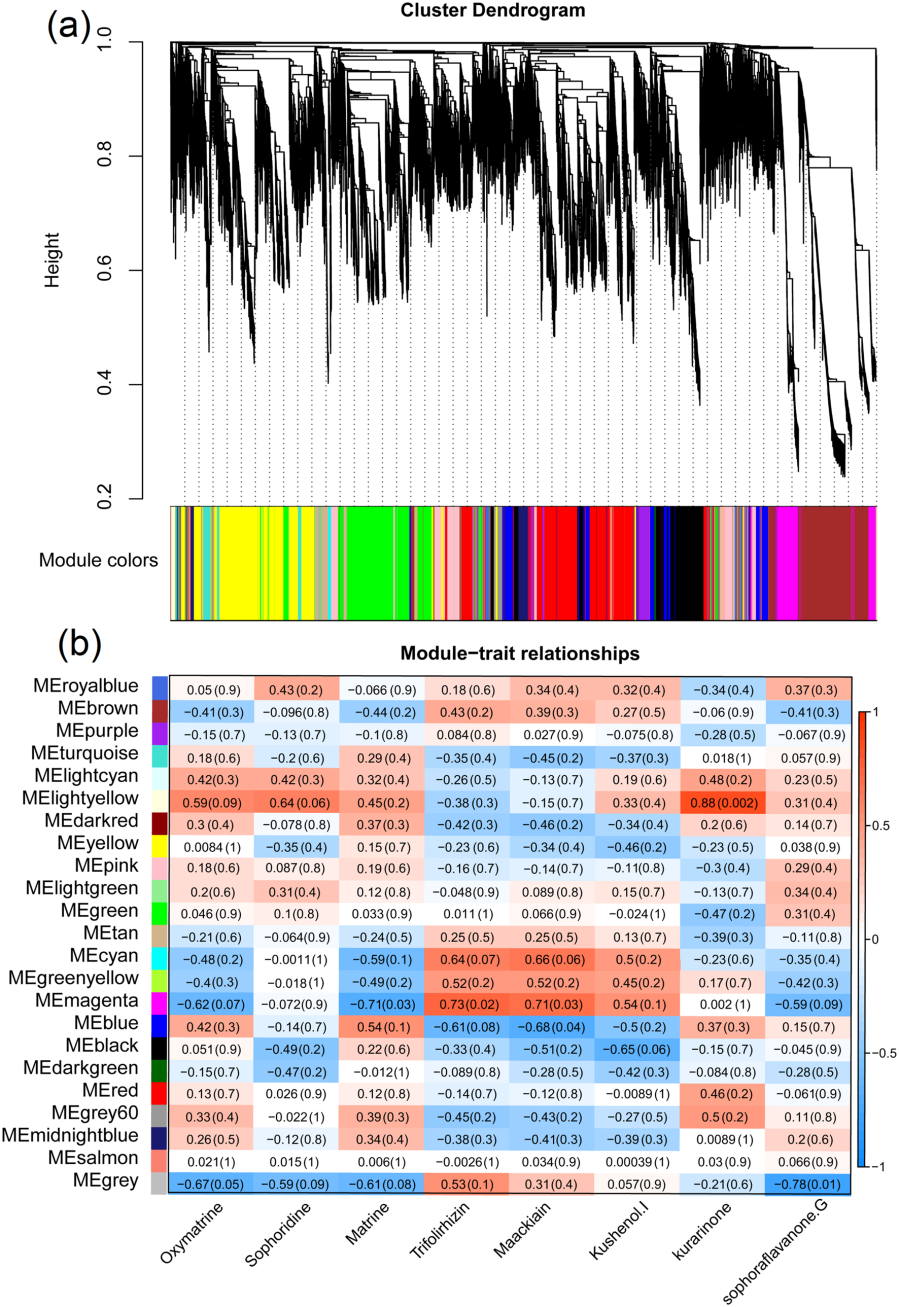
Co-expression profiles of all transcripts and active components in the root tissues of *S. flavescens*. (**a**) Hierarchical cluster tree showing co-expression modules in *S. flavescens*. (**b**) Module-components association in *S. flavescens*.

In *S. flavescens*, all transcripts were grouped into 23 unique modules, and two modules, namely, MEmagenta and MElightyellow, were positively correlated with active components (*P* < 0.05; Fig. 4a). MEmagenta was significantly and positively correlated with the contents of trifolirhizin (*R* = 0.73) and maackiain (*R* = 0.71; Fig. 4b). MElightyellow was positively correlated with the contents of kurarinone (*R* = 0.88).

### Analysis of the transcripts involved in the alkaloid and flavonoid biosyntheses in the root tissues of *S. flavescens*

**Figure 5:**
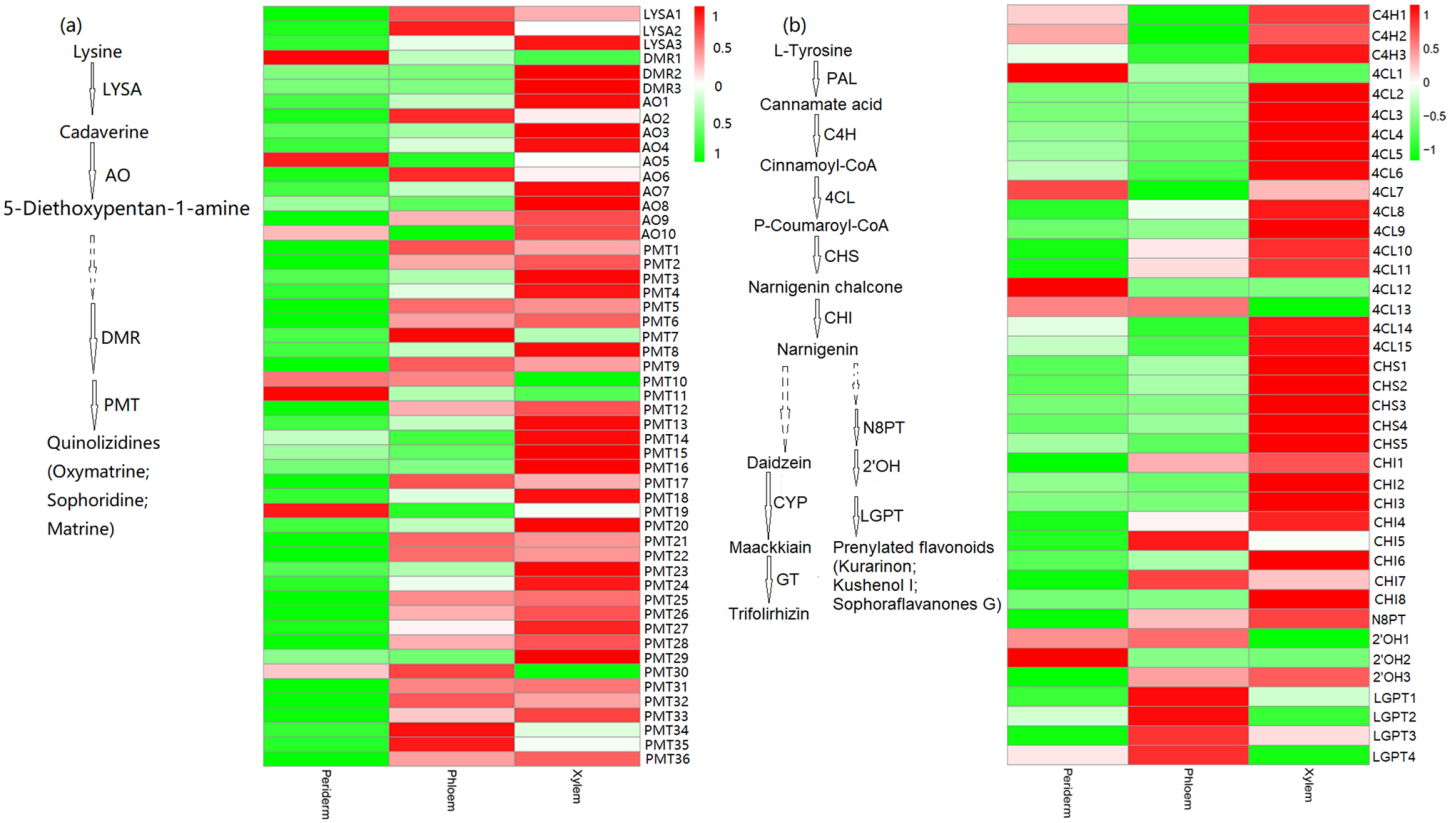
Heatmap of transcripts involved in the (**a**) alkaloid and (**b**) flavonoid biosyntheses in the roots of *S. flavescens*.

Key transcripts and enzymes resulted in various regulatory controllers in the alkaloid and flavonoid biosyntheses. The expression of most transcripts significantly differed (Fig. 5 and Supplemental file 2: Dataset S3, S4, and S5). In the alkaloid upstream biosynthesis pathway, 52 transcripts were selected to analyze expression profiles. Moreover, five (9.62%), 16 (30.77%), and 31 (59.62%) transcripts were expressed at the highest levels in the periderm, phloem, and xylem, respectively (Fig. 5a and Supplemental file 2: Dataset S3). One DMR transcript (DMR1), one AO transcript (AO2), and three PMT transcripts (PMT10, PMT11, and PMT19) showed the highest expression levels in the periderm. One LYSA (LYSA3), two DMR (DMR2 and DMR3), seven AO (AO1, AO3, AO4, AO7, AO8, AO9, and AO10), and 23 PMT (PMT2, PMT3, PMT4, PMT6, PMT8, PMT12, PMT13, PMT14, PMT15, PMT16, PMT18, PMT19, PMT20, PMT23, PMT24, PMT25, PMT26, PMT27, PMT28, PMT29, PMT31, PMT33, and PMT36) had the highest expression levels in the xylem. A total of 137 CYP transcripts participated in alkaloid synthesis were identified. Among them, 14 (10.22%), 52 (37.96%), and 71 (51.82%) transcripts were expressed at the highest levels in the periderm, phloem, and xylem, respectively (Supplemental file 1: Figure S3 and Supplemental file 2: Dataset S4). These results showed that the transcripts related to alkaloid synthesis were highly expressed in the xylem.

In the flavonoid biosynthesis pathway, 39 transcripts were selected to analyze expression profiles, wherein 5 (12.83%), 8 (20.5%), and 26 (66.67%) transcripts yielded the highest expression levels in the periderm, phloem, and xylem, respectively (Fig. 5b and Supplemental file 2: Dataset S5). Three 4CL transcripts (4CL1, 4CL7, and 4CL12) and one 2′OH transcript (2′OH2) exhibited the highest expression levels in the periderm. Three C4H (C4H1, C4H2, and C4H3), 11 CL (CL2, CL3, CL4, CL5, CL6, CL8, CL9, CL10, CL11, CL14, and CL15), 5CHS (CHS1, CHS2, CHS3, CHS4, CHS5), seven CHI (CHI1, CHI2, CHI3, CHI4, CHI6, and CHI8), and one 2′OH (2′OH3) yielded the highest expression levels in the xylem. These results showed that the transcripts related to flavonoid synthesis were highly expressed in the xylem.

### Correlation analysis of the active component contents and transcript expressions in *S. flavescens*

**Figure 6:**
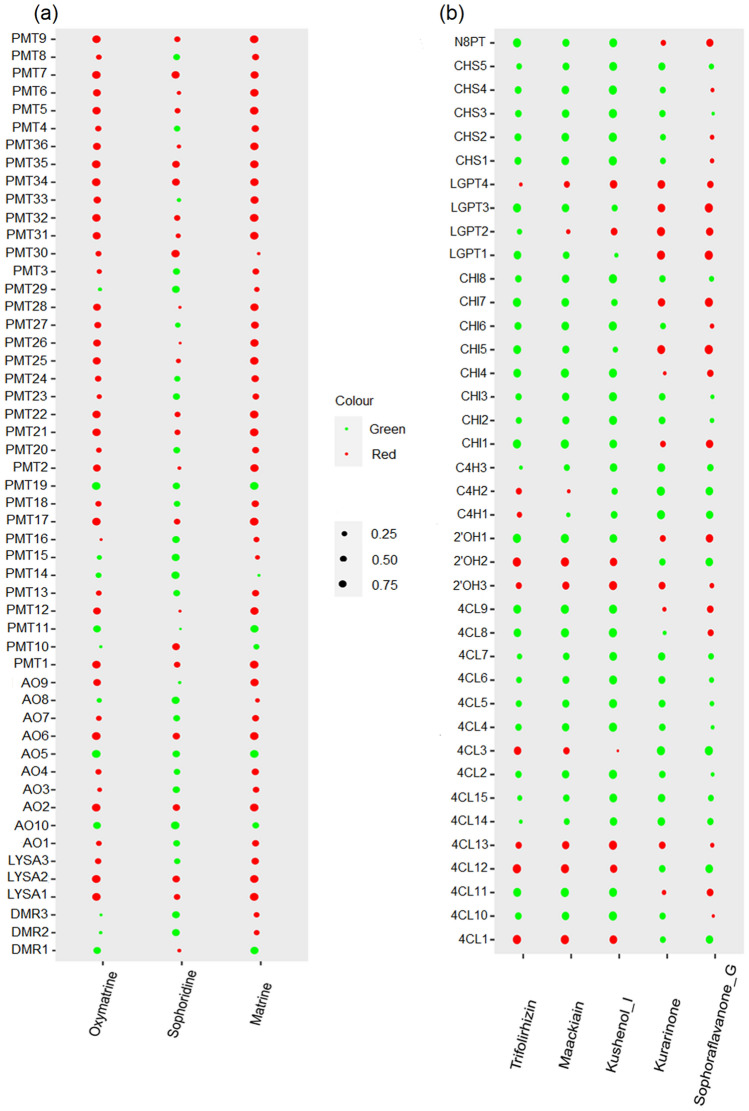
Pearson correlation bubble chart of the transcript expression and chemical component contents in *S. flavescens*: (**a**) alkaloids and (**b**) flavonoids. The size of the circle represents the correlation coefficient. The color red represents a positive correlation, whereas the color green represents a negative correlation.

The alkaloid synthetic pathway showed high and positive associations between component contents and transcripts: 16 transcripts with oxymatrine contents, two transcripts with sophoridine contents, and 24 transcripts for matrine contents (*R* > 0.8, *P* < 0.05, Fig. 6a). The expression levels of two LYSA (LYSA1 and LYSA2), two AO (AO2 and AO6), and 12 PMT (PMT1, PMT5, PMT7, PMT9, PMT17, PMT21, PMT22, PMT25, PMT31, PMT32, PMT34, and PMT35) transcripts were markedly and positively correlated with oxymatrine contents. The expression levels of two PMT (PMT7 and PMT30) transcripts were highly correlated with sophoridine contents. The expression levels of two LYSA (LYSA1 and LYSA2), three AO (AO2, AO6, and AO9), and 19 PMT (PMT1, PMT2, PMT5, PMT6, PMT7, PMT9, PMT12, PMT17, PMT21, PMT22, PMT25, PMT26, PMT28, PMT31, PMT32, PMT33, PMT34, PMT35, and PMT36) transcripts were markedly and positively correlated with matrine contents.

In the flavonoid synthesis pathway, a total of 3, 3, 3, 4, and 4 transcripts were highly and positively correlated with the contents of trifolirhizin, maackiain, kushenol I, kurarinone, sophoraflavanone G, respectively (*R* > 0.8, *P* < 0.05; Fig. 6b). The expression levels of two 4CL (4CL1 and 4CL12) and one 2′OH (2′OH2) transcripts were markedly and positively correlated with trifolirhizin and maackiain contents. The expression levels of two 4CL (4CL1 and 4CL13) and one 2′OH (2′OH1) transcripts were highly correlated with kushenol I contents. The expression levels of CHI5 and CHR1 transcripts were highly correlated with kurarinone and sophoraflavanone G contents.

## Discussion

In this study, the distribution of alkaloids and flavonoids demonstrated tissue specificity in *S. flavescens* roots. Transcript expression profiles also existed tissue specificity in the roots. The weighted gene co-expression network analysis (WGCNA) results confirmed that the pivotal transcripts regulated the distribution of alkaloids and flavonoids in the root tissues. This study will provide useful information for investigating the genetic and biochemical mechanisms of alkaloid and flavonoid syntheses.

Metabolite profiles revealed that the chemical components showed tissue specificity in the root tissues of *S. flavescens*. A total of 387 and 257 biomarkers were detected in positive and negative ion modes, respectively. The biomarkers detected in this study included many components of alkaloids and flavonoids. Alkaloids and flavonoids are the main chemical components of *S. flavescens*. They possess significant pharmacological effects, such as anti-tumor and anti-virus activities (matrine)^18^, hypoglycemic and hypolipidemic effects (oxymatrine)^19^, human colorectal cancer preventions (sophoridine)^20^, anti-proliferation (trifolirhizin)^21^, and inflammasome-activating effect (maackiain)^22^. Thus, the contents of alkaloids and flavonoids in the roots of *S. flavescens* were further quantitatively analyzed. Quantitative analysis results revealed that the contents of the total alkaloids and three alkaloids (oxymatrine, sophoridine, matrine) were higher in the phloem than those in the periderm and xylem. This finding was consistent with the reports of previous work^23^. The contents of trifolirhizin, maackiain, and kushenol I were the highest in the xylem, and the contents of kurarinone and sophoraflavanone G were the highest in the periderm. Four phenolic acid compounds (benzoic acid, caffeic acid, ferulic acid, and chlorogenic acid) and four flavonol compounds (kaempferol, catechin hydrate, epicatechin, and rutin) were higher in the aerial parts than the roots^13^. This uneven accumulation pattern of secondary metabolites may affect the rational use of medicinal plants. Understanding the molecular biological mechanism of the active components is of great significance.

The transcript expression in *S. flavescens* was tissue-specific. A total of 52 upstream transcripts and 137 downstream CYP transcripts involved in alkaloid synthesis were identified (FPKM ≥ 5), among which 59.62% and 51.82% were expressed at the highest levels in the xylem. In a previous study, the preferential expression of the gene for putative lysine/ornithine decarboxylase committed in the initial step of matrine biosynthesis was the highest in the leaf and stem^24^. The above finding evidentially indicated that the different expressions of these genes resulted in the uneven distribution of alkaloids. In the flavonoid compound biosynthesis pathway, 26 (66.67%) transcripts were highly expressed in the xylem. In a previous study, 41 transcripts were investigated and showed distinct expression profiles in different parts of *S. flavescens*^13^. The transcripts related to alkaloid and flavonoid biosynthesis in *S. flavescens* demonstrated organ-specific expression patterns, implying that they might have different physiological processes for biosynthesis, depending on the organ.

The correlation analysis results further showed that 28 and 12 transcripts were positively correlated with the contents of alkaloids and flavonoids, respectively (*R* > 0.8, *P* < 0.05). In the alkaloid biosynthetic pathway, the expression levels of LYSA1, AO6, and PMT transcripts were highly and positively correlated with the contents of alkaloids. In a previous study, seven enzyme genes involved in the alkaloid biosynthesis in *S. flavescens* were identified^25^. In the current study, three 4CL, three CHI, two 2′OH, and four CHR were highly and positively correlated with flavonoids in the flavonoid biosynthetic pathway. Phenylalanine ammonia-lyase, C4H, and 4CL were the three enzymes to form the substrate of the flavonoid compound p-coumaroyl-CoA^26^. Then, CHS catalyzed the formation of chalcone, and CHI catalyzed the chalcone formation of naringenin, a major metabolite in the synthesis of various flavonoids^27,28^. A previous study identified 13 enzyme genes involved in the flavonoid biosynthesis in *S*. *flavescens*^29^. In the current study, useful data for investigating the molecular and chemical information of the distribution of alkaloids and flavonoids in *S. flavescens* are provided.

## Materials and methods

All experimental research and field studies on plants, including the collection of plant material in this study, had complied with relevant institutional, national, and international guidelines and legislation.

### Plant materials

Three-year-old roots of *S. flavescens* were collected from Wenshan in Yunnan Province at their flowering stage. *S. flavescens* was cultivated with the standard operating procedures established by the Good Agriculture Practices ^30^. All roots were carefully washed and separated into three different parts: the periderm, phloem, and xylem (Supplemental file 1: Figure S1). The samples were divided into two parts for metabolite and transcriptome analyses.

### Metabolite analysis

All of the samples were dried and crushed, and 0.1 g of the powdered sample was weighed and mixed with 1.0 mL of pure methanol under vortex for 1 min and incubated at room temperature for 10 min^11^. The mixture was stored overnight at − 20 °C and centrifuged at 4000 g for 20 min. The upper layer was collected, filtered through a 0.22 µm filter, and transferred to a sample vial. The vial was injected into a column for UPLC-QTOF-MS analysis.

The UPLC-MS analysis was performed using a UPLC system (Waters, UK) coupled to an electrospray ionization-QTOF/MS apparatus (Waters, UK)^11^. A 100 mm × 2.1 mm C_18_ reversed-phase column (Acquity UPLC T3 column, Waters, UK) was used for UPLC separation, and the sample injection volume was 4 µL. The column temperature was kept at 35 °C, and the flow rate was maintained at 0.4 mL/min. The gradient was composed of water containing 0.1% formic acid (A) and acetonitrile containing 0.1% formic acid (B). The linear gradient was set as follows: 0–0.5 min for 5% B, 0.5–7 min for 5%–100% B, 7–8 min for 100% B, 8–8.1 min for 100%–5% B, and 8.1–10 min for 5% B.

A high-resolution tandem mass spectrometer TripleTOF5600plus (SCIEX, UK) was used to detect metabolites. The Q-TOF was operated in the positive and negative ion modes. The curtain gas was set to 30 PSI, the ion source gas1 was set to 60 PSI, the ion source gas2 was set to 60 PSI, and an interface heater temperature was set at 650 °C. Multivariate data analysis was performed using MetaboAnalyst 4.0 software (http://www.metaboanalyst.ca/)^11^. The PCA was performed to analyze the distribution of samples. One-way ANOVA was used to detect the difference of variance, and variance with FDR ≤ 0.05 was deemed as potential biomarkers. Variable VIP was used to evaluate the variable contribution.

### High-performance liquid chromatography-ultraviolet detection (HPLC-UV) analysis

The standards of oxymatrine, sophoridine, matrine, trifolirhizin, maackiain, kushenol I, kurarinone, and sophoraflavone G (purity ≥ 98.0%) were purchased from Shanghai Tauto Biotech Company (Shanghai, China). Their batch numbers were 16837–52–8, 6882–68–4, 519–02–8, 6807–83–6, 19908–48–6, 99119–69–4, 34981–26–5, and 97938–30–2, respectively.

The sample extracts were also used for alkaloid and flavonoid quantitative analyses. An Agilent HPLC-UV 1260 series system (Agilent, USA) equipped with a quaternary pump, automatic sampler, column compartment. A VWD was also employed. A 4.6 mm × 250 mm C_18_ reversed-phase column (with an inner diameter of 5 µm; Eclipse XDB, Agilent, USA) was used for separation, and the sample injection volume was set as 10 µL. The conditions for alkaloids were set as follows: column temperature of 30 °C, a flow rate of 1.0 mL/min, and a wavelength of 220 nm^31^. The gradient was composed of 80% acetonitrile (A), 10% ethanol (B), and 10% water (C). The conditions for flavonoids were set as follows: column temperature of 35 °C, a flow rate of 1.0 mL/min, and a wavelength of 295 nm ^32^. The gradient was composed of acetonitrile (A) and water (B), and the linear gradient was set as follows: 0–25 min for 19%–50% A, 25–30 min for 50%–70% A, 30–40 min for 70% A, and 40–50 min for 70%–40% A.

### RNA extraction and illumina sequencing

The total RNA was isolated from different tissues in accordance with the instructions indicated in a plant RNA isolation kit (BioTeke, Beijing, China). The quality of RNA was evaluated on 1% agarose gel, and RNA concentrations were determined with a Nanodrop 2000 spectrophotometer (Thermo Technologies). cDNA library construction and sequencing were performed in accordance with the standards of progress. First, mRNA was enriched from the total RNA by oligo (dT) magnetic beads and broken into short fragments^33^. Then, a random hexamer and RNA fragments were used to prime cDNA synthesis. After purification and connection with adapters, the cDNA library was constructed through PCR amplification. The length of an insert sequence was verified with an Agilent 2100 bioanalyzer system (Agilent Technologies, Santa Clara, CA, USA), and the library was quantified by an ABI Step One Plus real-time PCR system (Applied Biosystems, America). Finally, the qualified cDNA library was sequenced with an Illumina HiSeqTM 2000 system (Illumina Technologies).

### Transcriptome analysis

All raw reads were subjected to the cutadapt (v1.9) and fqtrim (v0.94) software following quality control to produce clean reads: (1) raw reads including adapter aequences and empty adapter were discarded; (2) reads including unknown N bases comprising more than 5% of the total length were filtered; (3) reads including low-quality bases that comprise more than 20% of the total length were discarded ^33^. Then, The indicaters of Q20% (sequencing error rate less than 0.01), Q30 (sequencing error rate less than 0.001), and GC% were calulated to evaluate the quality of clean reads. All the 150 bp pair-end RNA-Seq reads were submitted to NCBI (Accession number: PRJNA661972).

De novo assembly was performed in Trinity (v2.4.0) software using 150 bp pair-end reads with default parameters^34^. One assembly was performed using nine sequencing reads, and 58,327 transcripts were obtained. These resultant transcripts were searched against the NCBI nonredundant nucleotide (Nt) database, NCBI nonredundant protein (Nr), and SwissProt protein for functional annotation by using the BLAST algorithm with an E-value cutoff of 1e^−5^^35^. The functional categories of these unique sequences were further analyzed using the above databases and the KEGG database in BLAST and Blast2GO programs as previously reported in the literature^36–39^.

The clean reads were mapped to the reference by using Bowtie 2 (v2.2.6) to estimate the expression profiles of the transcripts^40^. The expression levels were calculated with the FPKM by using RSEM software (v1.3.1), and the bowtie parameter was set at mismatch 2^41^. The identification of DEGs was performed using the following criteria: fold change (FC) ≥ 2 and FDR ≤ 0.05. The candidate transcripts involved in the alkaloid and flavonoid biosyntheses were selected in accordance with previous reports and databases with FPKM values of the transcripts converted to log_10_ values (FPKM ≥ 5). They were visualized in a heatmap with pheatmap package (v1.0.12) in *R* to identify the different expression profiles among the three tissues^42^.

### Co-expression analysis

The WGCNA was used to analyze the relationships between transcript expressions and component contents with the *R* package (v3.2.5)^43,44^. The *R* package along with its source code and additional material are freely available at https://horvath.genetics.ucla.edu/html/CoexpressionNetwork/Rpackages/WGCNA/. The network construction and module detection method with default settings were used, including an unsigned topological overlap matrix. All parameters were set as defined: “soft_power = 22, TOMType = ‘unsigned’, minModuleSize = 30, reassignThreshold = 0, and mergeCutHeight = 0.25”. The *P*-value of 0.05 was set as the threshold for a significant correlation.

The candidate transcripts involved in the alkaloid and flavonoid biosyntheses were further selected in accordance with the annotation information to analyze the relationship of transcript expression with alkaloid and flavonoid contents. Pearson correlation coefficient of alkaloid and flavonoid contents with FPKM of transcripts were normalized and then calculated using SPSS (v17.0) software. Pearson correlation bubble chart was constructed with the *R* package (v3.5.0) to identify pivotal transcripts related to the contents of alkaloids and flavonoids^42^.

## Conclusion

To sum up, the alkaloids and flavonoids showed tissue specificity in *S. flavescens* roots. Gene expression profiles also showed tissue specificity. The metabolomes and transcriptomes systematically confirmed the pivotal transcripts regulating the distribution of alkaloids and flavonoids. This study elucidated the mechanism of alkaloids and flavonoids synthesis, accumulation, and transportation, which provide the basis for improving the production of alkaloids and flavonoids through genetic engineering. In addition, these genetic resources could provide comprehensive information on gene discovery, transcriptional regualtion, and variety selection for *S. flavescens*.

## Supplementary Information

Supplementary Information 1.

Supplementary Information 2.

## Data Availability

All of the transcriptome sequences were submitted to NCBI (Accession number: PRJNA661972).

## Abbreviations

UPLC-MS: Ultrahigh-performance liquid chromatography quadrupole time of flight-mass spectrometry
HPLC-UV: High-performance liquid chromatography-ultraviolet detection
PCA: Principal component analysis
ANOVA: One-way analysis of variance
VIP: Variable importance in projection
FDR: False discovery rate
KEGG: Kyoto encyclopedia of genes and genomes
FPKM: Fragments per kilobase of exon per million fragments
DEGs: Differential expression genes
NCBI Nr: NCBI non-redundant protein
WGCNA: Weighted gene co-expression network analysis
UGTs: UDP-glycosyltransferases
P450: P450-monooxygenase
C4H: Cinnamate 4-hydroxylase
4CL: 4-Coumaric acid coenzyme A ligase
CHS: Chalcone synthase
CHI: Chalcone isomerase
PAL: Phenylalanine/tyrosine anmonia-lyase
N8PT: Naringenin 8-dimethylallyltransferase
2′OH: 8-Domethylallylnaringenin 2′-hydroxylase
LGPT: Leachianone G 2′-dimethylallyltransferase
LYSA: Lysine decarboxylase
AO: Amine oxidase
DMR: Diaminopimelate decarboxylase
PMT: Probable methyltransferase

## References

[1] Zhao P, Zhang YJ, Yamamoto H, Yang CR (2004). Recent advance on the chemistry, bioactivity and biosynthesis of prenylated flavonoids from *Sophora flavecens*. Nat. Prod. Res. Dev..

[2] Gao, X.M. Traditional Chinese medicine. Beijing; China. *Trad. Chin. Med. Pub*. 1283 (2007).

[3] Sun J (2020). Effect of matrine sodium chloride injection on a mouse model combining disease with syndrome of human coronavirus pneumonia with cold-dampness pestilence attack on the lung. Acta Pharm. Sin..

[4] Yang MW (2020). Clinical effecacy of matrine and sodium chloride injection in the treatment of 40 cases of COVID-19. Chin. J. Chin. Mater. Med..

[5] Sun MY (2007). Novel antitumor activities of Kushen flavonoids in vitro and in vivo. Phytother. Res..

[6] Sun MY (2012). Antitumor activities of Kushen: Literature review. Evid.-Based. Complement Alternat. Med..

[7] Guan ZG, Wu SY, Guo BL, Wei HG, Wang YL, Yang X (2015). Comparative study on active ingredient content in different parts of *Sophora flavescens*. Mod. Chin. Med..

[8] Chen J, Sun F, Meng J, Wang SM, Liang SW (2013). Determination research of different parts in *Sophora flavescens*. Asia-Pac. Tradit. Med..

[9] Bai HR (2015). Localization of ginsenosides in Panax ginseng with different age by matrix-assisted laser-desorption/ionization time-of-flight mass spectrometry imaging. J. Chromatogr. B Anal. Technol. Biomed. Life Sci..

[10] Liang ZT (2015). Localization of ginsenosides in the rhizome and root of *Panax ginseng* by laser microdissection and liquid chromatography-quadrupole/time of flight-mass spectrometry. J. Pharm. Biomed. Anal..

[11] Wei GF (2020). Metabolomes and transcriptomes revealed the saponin distribution in root tissues of *Panax quinquefolius* and *Panax notoginseng*. J. Ginseng Res..

[12] He X, Fang J, Huang L, Wang J, Huang X (2015). Sophora flavescens Ait.: Traditional usage, phytochemistry and pharmacology of an important traditional Chinese medicine. J. Ethnopharmacol..

[13] Lee J (2018). Profiling of the major phenolic compounds and their biosynthesis genes in *Sophora flavescens* Aiton. Sci. World J..

[14] Wu Q (2010). Application of transcriptomics in the studies of medicinal plants. World Sci. Tech..

[15] Cao HZ (2015). Transcriptome analysis of methyl jasmonate-elicited *Panax ginseng* adventitious roots to discover putative ginsenoside biosynthesis and transport genes. Int. J. Mol. Sci..

[16] Gao W (2014). Combining metabolomics and transcriptomics to characterize tanshinone biosynthesis in *Salvia miltiorrhiza*. BMC Genomics.

[17] He B, Gu YC, Xu M (2015). Transcriptome analysis of *Ginkgo biloba kernels*. Front. Plant Sci..

[18] Chen J, Sun F, Jiang M, Wang SM, Liang SW (2013). Determinatin research of different parts in *Sophora flavecens*. Asia-Pac. Trad. Med..

[19] Liu Y, Xu Y, Ji W, Li XY, Sun B (2014). Anti-tumor activities of matrine and oxymatrine: literature review. Tumour. Biol..

[20] Guo CG, Zhang CF, Li L, Wang ZZ, Xiao W, Yang ZL (2014). Hypoglycemic and hypolipidemic effects of oxymatrine in high-fat diet and streptozotocin-induced diabetic rats. Phytomedicine.

[21] Wang R (2019). Sophoridine inhibits human colorectal cancer progression via targeting MAPKAPK2. Mol. Cancer Res..

[22] Lu X, Ma J, Qiu H, Yang L, Cao L, Shen J (2016). Anti-proliferation effects of trifolirhizin on MKN45 cells and possible mechanism. Oncol. Rep..

[23] Huh JW (2020). Maackiain, a compound derived from *Sophora flavescens*, increases IL-1β production by amplifying nigericin-mediated inflammasome activation. FEBS Open Bio.

[24] Han R (2015). Transcriptome analysis of nine tissues to discover genes involved in the biosynthesis of active ingredients in *Sophora flavescens*. Biol. Pharm. Bull..

[25] Zhang N, Yin MQ, Tan QQ, Wen YY, Wang YG, Wang JR (2019). SSR sites and gene function annotation analysis of *Sophora flavescens* transcriptome. JS. Agr. Sci..

[26] Weisshaar B, Jenkins GI (1998). Phenylpropanoid biosynthesis and its regulation. Curr. Opin. Plant Biol..

[27] Punyasiri PAN (2004). Flavonoid biosynthesis in the tea plant *Camellia sinensis*: properties of enzymes of the prominent epicatechin and catechin pathways. Arch. Biochem. Biophys..

[28] Tanner GJ, Francki KT, Abrahams S, Watson JM, Larkin PJ, Ashton AR (2003). Proanthocyanidin biosynthesis in plants. Purification of legume leucoanthocyanidin reductase and molecular cloning of its cDNA. J. Biol. Chem..

[29] Zhang FS, Wang QY, Pu YJ, Chen TY, Qin XM, Gao J (2017). Identification of genes involved in flavonoid biosynthesis in *Sophora japonica* through transcriptome sepuencing. Chem. Biodivers..

[30] Zhang B (2010). GAP production of TCM herbs in China. Planta Med..

[31] Geng F, Zhang N, Fang H, Li JM, Zhao X, Liu HY (2014). Metabonomic study on protective effect of Xiaoyao power for acute hepatic injury in rats. J. Chin. Med. Mater..

[32] Ma HY, Zhou WS, Chu FJ, Wang D, Liang SW, Li S (2013). HPLC fingerprint of flavonoids in *Sophora flavescens* and determination of five components. Chin. J. Chin. Mater. Med..

[33] Grabherr MG (2011). Full-length transcriptome assembly from RNA-Seq data without a reference genome. Nat. Biotechnol..

[34] Lieber MD (2013). novo transcript sequence reconstruction from RNA-seq: Reference generation and analysis with Trinity. Nnt. Protoc..

[35] Cao H, Nuruzzaman M, Xiu H, Huang J, Wu K, Chen X (2015). Transcriptome analysis of methyl jasmonate-elicited *Panax ginseng* adventitious roots to discover putative ginsenoside biosynthesis and transport genes. Int. J. Mol. Sci..

[36] Conesa A, Gotz S, Garcia-Gomez JM, Terol J, Talon M, Robles M (2015). Blast2GO: A universal tool for annotation, visualization and analysis in functional genomics research. Bioinformatics.

[37] Kanehisa M, Goto S (2000). KEGG: Kyoto Encyclopedia of Genes and Genomes. Nucleic. Acids. Res..

[38] Kanehisa M (2019). Toward understanding the origin and evolution of cellular organisms. Protein. Sci..

[39] Kanehisa M, Furumichi M, Sato Y, Ishiguro-Watanabe M, Tanabe M (2021). KEGG: Integrating viruses and cellular organisms. Nucleic. Acids. Res..

[40] Trapnell C, Hendrickson DG, Sauvageau M, Goff L, Rinn JL, Pachter L (2013). Differential analysis of gene regulation at transcript resolution with RNA-seq. Nat. Biotechnol..

[41] Li B, Dewey CN (2011). RSEM: Accurate transcript quantification from RNA-Seq data with or without a reference genome. BMC Bioinf.

[42] R Core Team. R: A language and environment for statistical computing. R Foundation for Statistical Computing, Vienna, Austria (2018)

[43] Langfelder P, Horvath S (2008). WGCNA: An R package for weighted correlation network analysis. BMC Bioinf..

[44] Langfelder P, Horvath S (2012). Fast R functions for robust correlations and hierarchical clustering. J. Stat. Softw..

