# Supplementary material for: Biosyntheses characterization of alkaloids and flavonoids in Sophora flavescens by combining metabolome and transcriptome
Source: Sci Rep. 2021 Apr 1;11:7388. doi: 10.1038/s41598-021-86970-0 (PMC8016917; doi:10.1038/s41598-021-86970-0)
Supplement: Supplementary file 1 — Supplementary Information 1. [file 41598_2021_86970_MOESM1_ESM.doc]

**Biosyntheses characterization of alkaloids and flavonoids in *Sophora flavescens* by combining metabolome and transcriptome**

Guangfei Wei^1^, Yongzhong Chen^1^, Xiaotong Guo^2^, Jianhe Wei^3^, Linlin Dong^1*^ and Shilin Chen^1**^

^1^ *Key Laboratory of Beijing for Identification and Safety Evaluation of Chinese Medicine, Institute of Chinese Materia Medica, China Academy of Chinese Medical Sciences, Beijing 100700, China*

^2^ *College of Agriculture, Ludong University, Yantai 264025, China*

^3^ *Hainan Provincial Key Laboratory of Resources Conservation and Development of Southern Medicine, Hainan Branch of the Institute of Medicinal Plant Development, Chinese Academy of Medical Sciences and Peking Union Medical College, Haikou 570311, China*

**E-mail addresses:**

Guangfei Wei: [gfwei@icmm.ac.cn](mailto:dll_aaa@163.com)

Yongzhong Chen: 1319633962@qq.com

Xiaotong Guo: guoxtina@126.com

Jianhe Wei: wjianh@263.net

*** Corresponding author**

TEL: (+86) 18911917789; fax: (+86) 1062899776; email: [lldong@icmm.ac.cn](mailto:lldong@icmm.ac.cn)

*** *Corresponding author**

TEL: (+86) 1057203877; fax: (+86) 1062899776; email: slchen@icmm.ac.cn

Present address: No.16 Nanxiaojie, Dongzhimennei Ave. Beijing 100700, China.

**Supplemental Information List**

**A. Supporting Figures**

Figure S1 Main root tissues in *S. flavescens*. Pe, Ph, Xy represent periderm, pholem and xylem, respectively.

Figure S2 Statistics of differential expression unigenes between two tissues of S. flavescens (FDR ≤ 0.05 & FC ≥ 2 ).

Figure S3 Heatmap of the average expression level of CYP transcripts related to alkaloids in the root tissues of S. flavescens (FPKM ≥ 5).


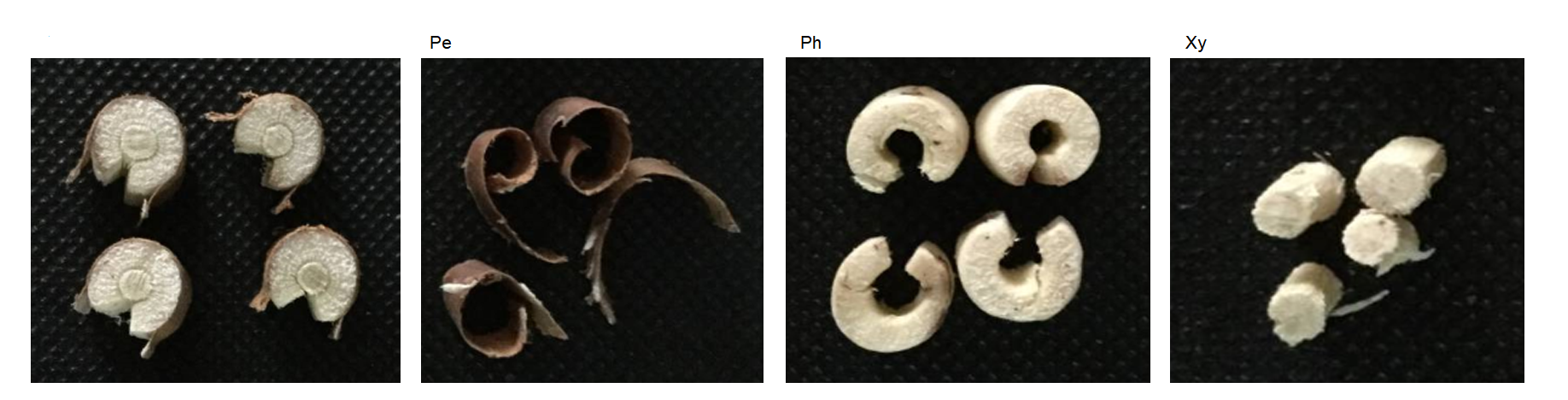


Fig. S1 Main root tissues in *S. flavescens*. Pe represents the periderm; Ph represents the pholem; Xy represents the xylem.


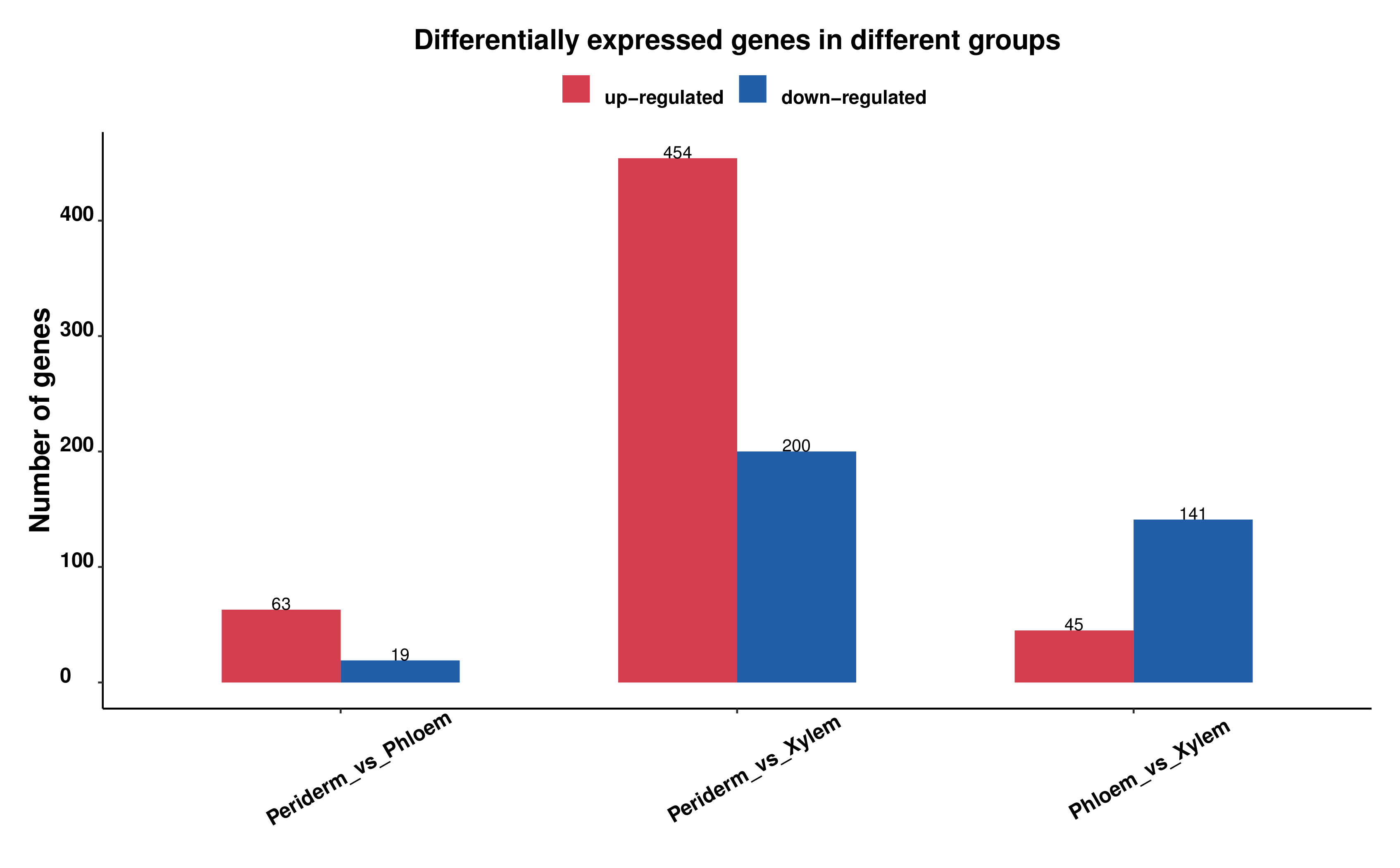


Figure S2 Statistics of differential expression unigenes between two tissues of S. flavescens (FDR ≤ 0.05 & FC ≥ 2 ).


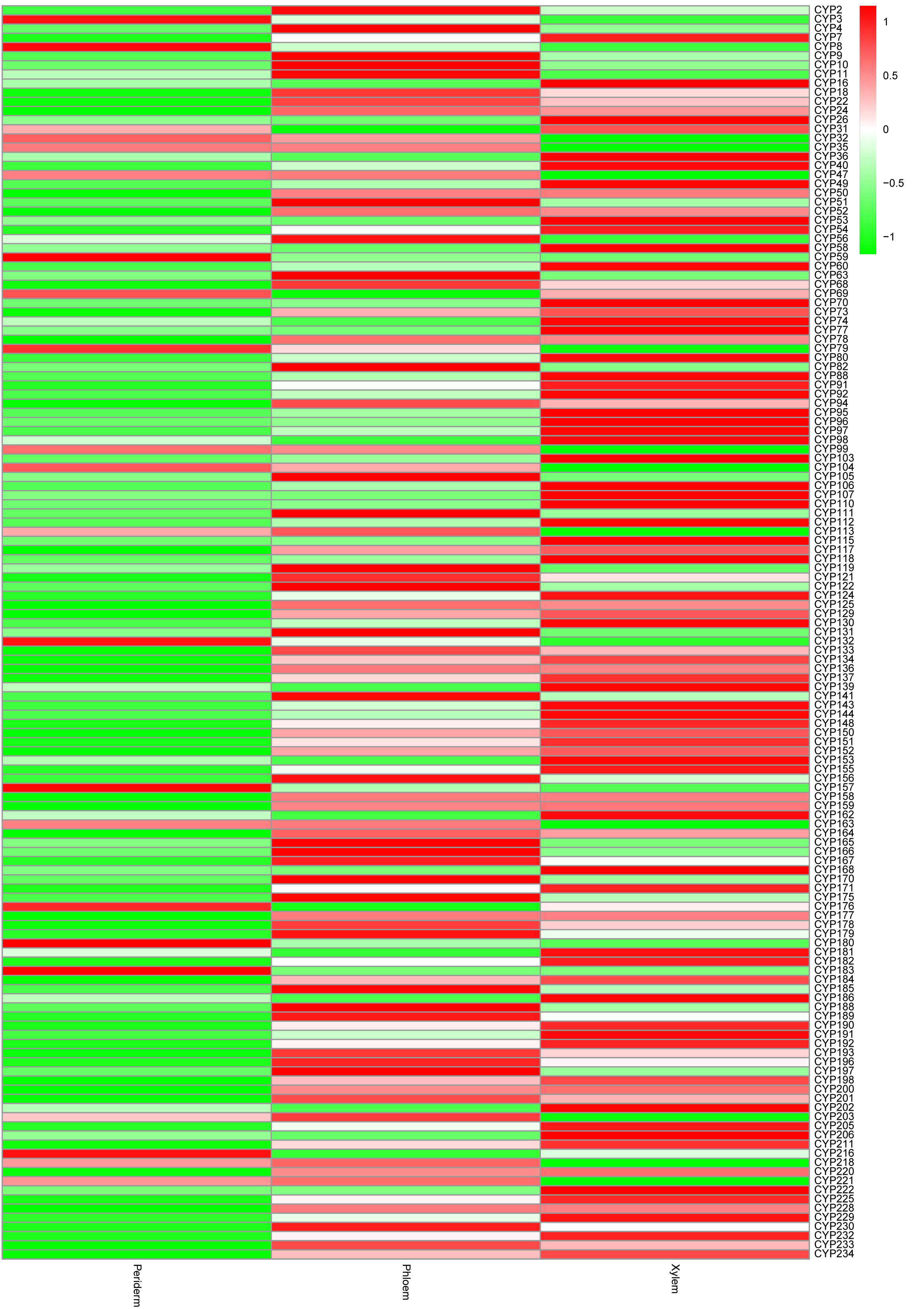
Figure S3 Heatmap of the average expression level of CYP transcripts related to alkaloids in the root tissues of S. flavescens (FPKM ≥ 5).
